# The role of mutation in the new cancer paradigm

**DOI:** 10.1186/1475-2867-5-9

**Published:** 2005-04-26

**Authors:** Richmond T Prehn

**Affiliations:** 1 Dept. of Pathology, University of Washington, Seattle, WA, USA

**Keywords:** carcinogenesis, mutation, cloning, epigenetic, differentiation, cancer regression

## Abstract

The almost universal belief that cancer is caused by mutation may gradually be giving way to the belief that cancer begins as a cellular adaptation that involves the local epigenetic silencing of various genes. In my own interpretation of the new epigenetic paradigm, the genes epigenetically suppressed are genes that normally serve in post-embryonic life to suppress and keep suppressed those other genes upon which embryonic development depends. Those other genes, if not silenced or suppressed in the post-embryonic animal, become, I suggest, the oncogenes that are the basis of neoplasia.

Mutations that occur in silenced genes supposedly go unrepaired and are, therefore, postulated to accumulate, but such mutations probably play little or no causative role in neoplasia because they occur in already epigenetically silenced genes. These mutations probably often serve to make the silencing, and therefore the cancer, epigenetically irreversible.

## The present paradigm

For the past half century, most cancer investigators have thought, beyond any reasonable doubt, that each cancer has its origin in a single cell that undergoes mutations to give that cell a cancer phenotype or at least to set that cell and its progeny on the path to forming an overt cancer [1]. This paradigm has been fostered by the ever increasing awareness that cancers often harbor large numbers of mutant genes and chromosomal abnormalities and that many carcinogens are mutagens. The presence of large numbers of mutations has led to the hypothesis that cancers may often progress because they contain a mutator phenotype [2].

There is seemingly convincing evidence that many cancers are of clonal origin, ie., they apparently develop from a single cellular progenitor [3], a fact suggestive of a mutational origin. It is also true that certain hereditary cancers are associated with particular mutant genes. For example, In the rare disease xeroderma pigmentosum, the inherited genetic aberration increases susceptibility to UV-induced mutagenesis and to skin cancer by as much as 1000 fold [4]. This increase in tumor incidence, consequent upon a particular inherited background genotype, is not really relevant to the question of whether or not mutation is the "cause" of cancer. The background genotype obviously plays a great role in determining the frequency with which cancer emerges, but for our purposes the cause is whatever it is that determines which particular cell or group of cells, among the many of the same genotype, shall proliferate to form a neoplasm.

## The need for a new paradigm

A few observers have persisted in pointing out that there are certain facts that are difficult to reconcile with the current mutational paradigm [1,5-9]. None of the arguments is conclusive- there would be no debate if that were the case- but in aggregate they do make a strong case for questioning the prevalent paradigm.

One observation that suggests that the mutation paradigm might be wrong is the fact that, among chemical carcinogens, some fifty percent of those examined were, at least within the sensitivity limits of the test (Ames test), seemingly non-mutagenic, at least in E. *coli *[10]; more importantly, although there is some correlation between the mutagenic and carcinogenic potencies of chemical carcinogens, there are potent chemical carcinogens, such as TCDD (2,3,7,8-tetrachlorodibenzo-*p*-dioxin) that are not mutagens even as judged by the most modern methods available[11].

Furthermore, a thin film of apparently any smooth material, introduced under the skin of a mouse, can induce a sarcoma, but if the surface of the film were appropriately roughened, tumor is not induced [12]. This phenomenon suggests that a mere tissue disturbance, a disturbance that is unlikely to be mutagenic, can induce neoplasia. This notion is consonant with the purported roles of inflammation [13] and of trauma [14] in the genesis of many cancers. In chemical carcinogenesis, a recent study has shown that the carcinogen need not even be applied directly to the cells that undergo transformation. Thus, application of the carcinogen to the stroma of the mammary fat-pad in the rat could induce epithelial cancers in untreated mammary cells that were subsequently exposed to that previously treated stroma [15]; since the carcinogen had long since disappeared, a disturbed stromal-epithelial interaction was apparently critical. Other analogous experiments have been reviewed [15]

An early observation that called the mutation hypothesis into some question was that when a carcinogen was applied to a tissue or to a culture of supposedly normal cells, persistent change could be observed in most or even in all of the targets. Thus, for example, exposure of mouse cells in culture to a carcinogenic chemical produced, in 100% of the cells, a change that made them hypersusceptable to transformation [16]. Furthermore, the cells became resistant to the toxicity of the carcinogen [5,17,18]. Mutation is usually thought of as a rather random and sporadic process very different from a phenomenon in which virtually all target cells show the same adaptive change.

A large number of analogous observations have been reviewed [8]. These studies suggest that cancer may usually be initiated in a broad field of tissue that undergoes a general adaptive change rather than being initiated by rare mutational changes [5].

Perhaps the most persuasive observations suggesting that some change other than mutation underlies the induction of cancer are observations concerning regressions. If regression occurs via a return of cancer cells to a normal non-cancerous phenotype, it becomes difficult to argue that the lesion had been the result of mutation. Clinically, cancer regression is a well documented fact, albeit that it occurs in a very small percentage of well-established clinical cases. It is more common in childhood cancer, most notably in neuroblastoma, but can be observed sporadically in a vast variety of neoplasms. Most of these regressions are probably caused by a change to a more normal phenotype, a differentiation rather than cell killing. This process of differentiation seems clear in the case of neuroblastoma [19].

Painting the mouse skin with a chemical carcinogen can result in numerous papillomas. Few of these progress to become malignant; most regress. It has been shown in the mouse-skin system that the regression can occur in the probable absence of any anticancer immunity [20]. In the analogous liver system, reversion of the initial lesions to a more normal phenotype is well documented [5]. Regressions are characteristic of only the initial papillomas; after some progression of a lesion, if such occurs, regression of that lesion becomes less common. Be it noted that it is probable that many epithelial tumors in mammals, but apparently not all [21], are preceded by small, benign, focal hyperplasias, most of which do not progress and many of which may regress.

Regression toward a normal phenotype has also been demonstrated in some other experimental systems; most notably, by the injection of embryonal carcinoma cells into a developing blastocyst [22,23]. Hepatocarcinoma cells have also been normalized in the environment of the liver [24]. Similarly, melanoma was regulated by embryonic skin [25]. Recently, Hochedlinger et al. reported reprograming of a melanoma genome by nuclear transplantation into an enucleated egg [26]. A variety of other methods have also been able to induce reprogramming and differentiation in cancer cells [27,28].

It could be argued that, although in all these cases the cancer genes were able to produce an apparently normal phenotype, perhaps a mutated genome nonetheless remained, its oncogenic potential nullified by still unknown homeostatic devices. However, in the Illmensee and Mintz experiment, it was reported that embryonal carcinoma cells placed in the blastocyst sometimes gave rise, within the resulting mouse, to germ cells of cancer origin which could be used to produce an apparently normal second generation mouse! The authors concluded "that teratocarcinogenesis entails changes in gene function rather than gene structure" [23].

## The new paradigm

The new paradigm that seeks to explain these various studies has been most recently expounded by Soto and Sonnenschein who call their version the TOFT or "tissue organization field theory "[1]. The essence of the new paradigm is the concept that disturbances in cell to cell signaling rather than mutation result in the heritable changes in gene expression that are the cause of cancer. What follows is my own interpretation of the new paradigm stressing possible reasons why some cancers, while probably not caused by mutation, exhibit so many mutations.

### (A).......Starting premise: Cancer is initiated by a loss of gene function

Soto and Sonnenschein [1] best articulated the fact that the default condition in single-celled species and in cells separated in tissue culture is not quiescence, but proliferation. Cells seem to proliferate unless inhibited by being part of a multicellular organism. Thus, it follows that when cells proliferate in a neoplasm, it is probably because the natural inhibitors to cellular proliferation found in multi-celled creatures are, for one reason or another, defective. In the neoplasm, the normal inhibition to proliferation has been, to a large degree, removed. Being impressed with the extent to which cancers and embryos share characteristics, I am inclined to believe that the critical genes that lose expression are often among those whose function is to silence those genes that specify embryonic development. The latter genes I will, for convenience, term ED for *embryo development *genes and the former I will call SED for *suppressors of embryo development *genes. It is in the silencing of SED genes that A. Knudsen's two hit theory may be relevant [29]. Because the SED genes are themselves naturally silenced in the embryo, embryonic growth and development can proceed. As the animal ages, various SED genes, that were unexpressed in the embryo, gradually become expressed according to the programmed schedule of embryogenesis. The genes specifying embryonic growth, the ED, are, thereby, gradually silenced. When and if, at some later time in life and at some particular site, the SED genes, because of some malfunction, are again silenced, corresponding ED genes might again be expressed. Such reexpression, now as oncogenes, might lead locally to cellular proliferation and possible neoplasia. Thus, a neoplasm could be considered a post-embryonic, bizarre, local recapitulation of one or more facets of embryogenesis caused by the pathologic reexpression, in the post-embryonic animal, of normal embryo development genes (ED).

If this thesis is correct, apparent "oncogenes" are probably ED genes whose normal function, when expressed in the embryonic animal, is to support embryonic development, but if pathologically reexpressed in post-embryonic life may result in neoplasia. Thus, meduloblastoma, a common brain tumor in childhood, is apparently dependent upon the reactivation and perhaps actual amplification of *OTX2*, a so-called oncogene that is an ED gene essential for normal brain development, but whose expression is ordinarily suppressed in later life [30]. Suppression of *OTX2 *in meduloblastoma was apparently therapeutic [30], just as in other studies, inactivation of the oncogene *MYC *restored hepatocellular carcinoma to a normal phenotype [31]. Conversely, "tumor suppressor genes" appear to be SED genes whose inactivation may promote tumor growth by allowing greater expression of ED genes.

There is much evidence to show that among the bizarre chromosomal abnormalities often seen in cancers, there are some, such as particular translocations, that are associated with a particular type of neoplasm. The best studied of these may be the translocation that results in the Philadelphia chromosome and chronic myelogenous leukemia. It is clear that the chromosomal breakpoints tend to be associated with the expression of particular oncogenes [32]. The new paradigm does not shed light upon whether the oncogene expression precedes or follows the chromosomal breakage, but does suggest that some critical epigenetic disruption in cell-to-cell signaling probably antedated both.

### (B)........DNA repair does not occur or is slow among silenced genes

There is experimental evidence for this phenomenon, at least in a transgene [33]. It seems entirely reasonable that an organism would not expend much energy to repair unexpressed or silenced genes. Because of this normal lack of DNA repair, I postulate that mutations will tend to accumulate, at each cellular replication [34], among those genes silenced during carcinogenesis; ie., mutations will accumulate among silenced SED genes whose normal function in the adult had been to inhibit the corresponding ED genes that specify embryonic-type growth and development. Thus, my interpretation of the new paradigm suggests that most mutations in cancer are the result of the lack of repair in silenced SED genes; the mutations, for reasons to be explained shortly, are postulated to be, at least in most cases, the result rather than the cause of the silencing. Unrepaired mutations among unexpressed genes might sometimes be numerous and might even simulate the action of a mutator phenotype without the necessity of a mutation in any of the normal DNA repair mechanisms. Mutations may also be common in unexpressed genes in normal adult cells, but these may remain unnoticed unless revealed in a cancer.

One corollary is the prediction that some tumors may not, in actuality, have many more mutations than does the specific normal tissue of origin. There is a dearth of studies actually comparing the mutation frequencies in tumors with the frequencies in comparably treated but non-tumorous parental tissues. However, the data in one recent paper suggest that the frequency of mutations in tumors may often be no greater than that in comparably treated but non-tumorous tissues of the same type as that in which the tumor had originated [35]. These data argue against the etiological importance of most of the mutations that occur in neoplasms and suggest, additionally, that a mutator phenotype may not be common. However, the postulated lack of DNA repair in silenced genes suggests that when tumors, perhaps late in their progression, do exhibit numerous mutations, these probably occurred in previously silenced genes.

### (C).........Mutations will eventually "hard wire" the silenced genes

The longer a cancer has been in existence and the larger the lesion, the greater, presumably, will be the number of mutations. The mutations are postulated to confirm and solidify rather than cause the neoplastic state; these mutations will tend to "hard wire" and make irreversible the lack of expression in epigenetically silenced SED genes within a tumor. Gene expression in neoplasia, at least in Morris hepatomas, has indeed been observed to become relatively fixed and unresponsive to environmental signals [36]. Although these apparently diploid hepatomas were induced and selected to have the least change from normal that a transformed cell could possibly have, "the Morris hepatomas have a common tendency to be resistant to various combinations of inductions and repressions. Each line tends to be more or less frozen...."[37]. Probably, because of mutations in the newly silenced genes, a neoplasm will, over time, become less and less susceptible to any regression that might involve an epigenetically-induced return to a more normal pattern of gene expression.

This formulation offers a possible insight into the well documented tendency of various types of aberrant growths and neoplasms to undergo spontaneous regressions in the infant and for malignancies in early childhood to be surprisingly amenable to treatment. It seems reasonable to propose that the processes of embryonic growth and development gradually subside as an infant matures; presumably, this subsidence is caused by the gradually increasing expression of SED genes which would silence the expression of the ED genes upon which normal development depends. Neoplasia, in my interpretation of the new paradigm, is postulated to be dependent upon the locally inappropriate and untimely expression of ED genes consequent upon the silencing of their corresponding SED genes. However, as the infant ages, the suppressor genes (SED) are programed to gradually increase their expression; gradually the inappropriately expressed ED genes within the tumor might therefore be suppressed and thus make a childhood neoplasm, such as a neuroblastoma [19], surprisingly likely to lose its neoplastic qualities and undergo differentiation.

It has been observed that the newborn animal may be more susceptible to chemical carcinogens than is the adult [38]. Could this phenomenon also be related to the postulation that SED genes have not as yet, in the newborn, become fully expressed and that their expression is consequently more easily blocked by a carcinogen than would be the case at a later age?

In a possibly related observation, it has been observed that transplantation of the nucleus of a frog-kidney carcinoma-cell into an enucleated frog egg commonly supported normal development through the swimming tadpole stage [39]. No adult frogs were obtained. Apparently, the ED genes of the cancer cell nucleus lacked mutations that would have prevented embryonic development, but the presumed "hardwired" changes (mutations) in the silenced suppressor genes (SED) may have prevented the transition to the normal adult condition of silenced and unexpressed ED. In other words, the carcinoma nucleus was competent for either embryo or tumor growth, but was apparently incompetent for normal adult functioning, a set of facts that seems to fit perfectly with the thesis I have been advancing.

Parenthetically, random mutations in silenced genes may be the cause of the relative difficulty experienced in cloning adult somatic cells for reproductive purposes; most of the developmental genes (ED) in the adult cells would have been silent for long periods of time and thus may sometimes have acquired too many unrepaired mutations.

### (D)........The loss of expression among the genes that suppress developmental genes, is seldom caused by mutation

According to my interpretation of the new paradigm, the loss of expression among SED genes is usually the result of epigenetically-induced disruptions of normal cell to cell signals. That the signaling disruptions are probably not caused by mutation is inferred primarily by the previously noted tendency of early tumors to undergo regression via a return to a normal pattern of gene expression, a phenomenon which would be more difficult to explain were the disruptions mutational. The correct signals are supposedly essential, in the adult, for ensuring transcription in those genes (SED) that normally suppress developmental genes (ED).

## Objections to the new paradigm

A possible objection to the new paradigm is the fact that there is much evidence that each cancer may often be clonally derived from a single cell [3,40,41]. On its face, this evidence suggests mutation. However, there are other reasonable explanations for apparent clonality. The new paradigm suggests that cancer supposedly originates via adaptive epigenetic changes within a tissue; however, the arisal of focal papillomas suggests that the adaptive changes are not uniformly great among all the cells of that tissue. Thus, some clones may achieve a competitive advantage (based upon epigenetically-induced differential gene expression) and this competitive advantage might eventually and erroneously simulate an origin from a single cell. Supporting this hypothesis is the observation that mouse skin papillomas induced by initiation-promotion appear to be clonal while those induced by carcinogen alone are multiclonal [42] (unless induced by a very small dosage of carcinogen [3]).

## Conclusion

The new paradigm states that cancer is usually caused by epigenetic disturbances that interfere with cell to cell signaling. My own view of the new paradigm is that this interference probably silences, in a stable and heritable way, genes (SED) whose function, when expressed, had been to repress various genes (ED) responsible for aspects of embryonic development. A neoplasm is thus considered, in my formulation, to be caused by the local reexpression of normal embryonic genes (ED), genes that should have been expressed only at an earlier time and/or a different place.

I also propose that mutations tend to go unrepaired in those genes already silenced by epigenetic mechanisms; these mutations are postulated to accumulate without phenotypic consequence. Silenced genes, both in normal tissues and in tumors, presumably beget mutations, but these, according to my interpretation of the new paradigm, seldom play a significant role in the etiology of cancer. However, such mutations may act to "hard wire" the silenced suppressor genes (SED); this may result in the irreversible expression of embryonic genes (ED) which, in a cancer, are called oncogenes and which drive cancer development. This formulation explains why cancers tend to become with time progressively less likely to regress and progressively more difficult to treat.

Although my formulation of the new paradigm is indubitably a simplistic view of a very complex process, it seems congenial with present information and, I believe, may actually reflect, to some extent, the true reality.
